# Effects of preoperative calcium levels and parathyroidectomy on renal function in primary hyperparathyroidism: a retrospective study

**DOI:** 10.3325/cmj.2020.61.33

**Published:** 2020-02

**Authors:** Seher Kir, Cafer Polat

**Affiliations:** 1 Internal Medicine Department, Faculty of Medicine, Ondokuz Mayıs University, Samsun, Turkey; 2 General Surgery Department, Faculty of Medicine, Ondokuz Mayıs University, Samsun, Turkey

## Abstract

**Methods:**

This retrospective study enrolled 71 patients who underwent parathyroidectomy for PHPT in the General Surgery Department at Ondokuz Mayis University Hospital from 2010 to 2018. All patients were histopathologically diagnosed with parathyroid adenoma. Total serum calcium, serum creatinine, serum intact parathyroid hormone (PTH), and serum 25-hydroxyvitamin D_3_ (25(OH)D_3_) were measured before and 3-6 months after surgery. Patients were assigned to the low eGFR group (60-90 mL/min/1.73 m^2^) or normal eGFR group (≥90 mL/min/1.73 m^2^) and to the low calcium group (≤11.2 mg/dL) or high calcium group (˃ 11.2 mg/dL).

**Results:**

In the low eGFR and high calcium group, there were significantly more patients with hypertension and older age. In the normal eGFR and high calcium group, eGFR was significantly reduced after surgery. Independent predictors of eGFR change after surgery were age, pre-parathyroidectomy calcium, and pre-parathyroidectomy eGFR.

**Conclusions:**

After surgery, patients with low eGFR had preserved renal function, whereas those with normal eGFR had decreased renal function. Mild renal dysfunction in PHPT was associated with older age, hypertension, and high calcium levels.

**Aim** To assess the effects of preoperative calcium levels and parathyroidectomy on estimated glomerular filtration rate (eGFR) in patients with primary hyperparathyroidism (PHPT) with mild renal dysfunction or normal renal function.

## 

Primary hyperparathyroidism (PHPT) is a common endocrine disorder characterized by hypercalcemia and high or inappropriately normal parathyroid hormone (PTH) levels (1). It is most frequently caused by a single parathyroid adenoma, followed by parathyroid gland hyperplasia and parathyroid carcinomas (2). Patients with PHPT experience renal complications such as nephrolithiasis and hypercalciuria (3). Furthermore, in these patients high serum calcium and PTH levels may negatively affect the renal function (3). Although chronic renal failure mainly leads to secondary hyperparathyroidism, about 16%-17% of patients with PHPT have low glomerular filtration rates (eGFR<60 mL/min/1.73 m^2^) (1,4). Thus, a decrease in eGFR can be expected during a course of untreated PHPT. An eGFR below 60 mL/min/1.73 m^2^ is a surgical indication for parathyroidectomy (5). While some studies (5,6) reported that parathyroidectomy improved renal function in PHPT patients with eGFR below 60 mL/min/1.73 m^2^ pre-surgery, others reported no effect (7-12). However, the effect of parathyroidectomy on eGFR in patients with eGFR≥60 mL/min/1.73 m^2^ is not clear due to the scarcity of the studies on this issue (1,4,5,13,14). Moreover, the existing studies differ in terms of patient selection and methodology and report different factors affecting GFR. The aim of the present study was to assess the effects of preoperative calcium levels and parathyroidectomy on renal function in patients with PHPT with mild renal dysfunction or normal renal function.

## Patients and methods

### Study population

We retrospectively reviewed the records of all patients who underwent surgery due to PHPT in the General Surgery Department of the Ondokuz Mayis University Hospital from 2010 to 2018. All patients had histologically and pathologically confirmed parathyroid adenoma. None of the patients had advanced kidney failure (eGFR<60 mL/min/1.73 m^2^). All the surgeries were performed by the same surgical team. The pre-parathyroidectomy eGFR level ≥60 mL/min/1.73 m^2^ was used to eliminate the effects of moderate to severe renal insufficiency on the PTH hormonal axis.

Inclusion criteria were age of 18 years and older, complete medical records with a full patient history, available results of a physical examination, and available values of total serum calcium, serum creatinine, serum intact PTH, and serum 25-hydroxyvitamin D3 (25(OH)D_3_) before and 3-6 months after parathyroidectomy. Exclusion criteria were the diagnosis of multiple endocrine neoplasia type 1 and 2 or any active malignancies, including thyroid or parathyroid carcinomas diagnosed during parathyroidectomy, and thyroid or hepatic dysfunction. The final sample consisted of 71 patients (91.5% women).

The patients were divided into two groups according to Kidney Disease: Improving Global Outcomes guidelines (15): those with a mild decrease in eGFR (low eGFR group: 60-90 mL/min/1.73 m^2^) and those with a normal or increased eGFR (normal eGFR group: ≥90 mL/min/1.73 m^2^). One of the surgical criteria for PHPT is an increase in serum calcium level 1 mg/dL above the upper reference limit. Therefore, the patients were divided into low calcium group (≤11.2 mg/dL) and high calcium group (˃ 11.2 mg/dL) for the purpose of assessing the effect of calcium levels on eGFR.

This study was approved by the Medical Ethics Committee of Ondokuz Mayis University (OMU-KAEK 2018/296). The patients gave informed consent for the testing as part of their regular medical care. Demographic data, medical histories, histological examinations, laboratory studies, and imaging were obtained from the hospital database.

### Biochemical evaluation

Serum calcium and creatinine were measured with colorimetric and spectrophotometric methods by using Roche Cobas 8000 modular analyzer (Roche Diagnostics, Mannheim, Germany). Serum intact PTH was measured with electrochemiluminescence immunoassay method by using Roche Cobas E170 modular analyzer (Roche Diagnostics). Serum 25(OH)D_3_ was measured with high performance liquid chromatography (HPLC) by using Agilent 1100 Series (Chromsystem Diagnostics, Munich, Germany). Intra-assay and inter-assay coefficient of variations were 4.8% and 6.3%, respectively.

eGFR was assessed with the Chronic Kidney Disease Epidemiology Collaboration creatinine equation (15): 141 × min (SCr/ҡ, 1)^ᵅ^ × max(SCr/ҡ, 1)^1.209^ × 0.993^Age^ ( × 1.018 if female), where SCr is serum creatinine (in mg/dL), ҡ is 0.7 for women and 0.9 for men, ᵅ is -0.329 for women and -0.411 for men, min is the minimum of SCr/k or 1, and max is the maximum of SCr/k or 1.

### Statistical analysis

The normality of distribution was tested either by Shapiro-Wilk or Kolmogorov-Smirnov tests according to the number of participants in the total group and subgroups. The results are presented as mean ± standard deviation or median and interquartile range (IQR). The significance of differences between the groups for unpaired samples (eGFR and calcium groups) was tested with the Mann-Whitney U test or independent sample *t* test and for paired samples (pre-post PTX comparisons) with the Wilcoxon’s test or paired *t* test, where applicable. The significance of differences between the categorical variables was assessed with the chi square test or Fisher exact test, where applicable. The correlation between continuous variables was assessed with the Pearson correlation test or Spearman correlation test, where applicable. Multiple linear regression was used to evaluate independent predictors of eGFR change. Backward elimination method in multiple linear regression was used to predict the most significant model that explains the factors related to eGFR change. The level of significance was set at *P* ≤ 0.05. The analysis was conducted with SPSS, version 25.0 (IBM Corp., Armonk, NY, USA).

## Results

The mean age was 52.8 ± 12.1. Hypertension was present in 40.8%, diabetes mellitus (DM) in 16.9%, and nephrolithiasis in 18.3% patients. The mean eGFR was 97.7 ± 19.5 mL/min before parathyroidectomy and 95.5 ± 19.8 mL/min after parathyroidectomy. Serum calcium and PTH significantly decreased after parathyroidectomy, serum creatinine and 25(OH)D_3_ significantly increased, while eGFR did not change significantly (Table 1).

**Table 1:** Clinical data of primary hyperparathyroidism patients before and after parathyroidectomy*

	Before parathyroidectomy, mean±SD or median (IQR)	After parathyroidectomy, mean±SD or median (IQR)	P
**Age, years**	52.8 ± 12.1		
**Serum creatinine** (RR: 0.4-1.4 mg/dL)	0.69 ± 0.16	0.72 ± 0.18	0.032^†^
**Serum calcium** (RR: 8.8-10.2 mg/dL)	11.3 ± 0.75	9.5 ± 0.41	<0.001^†^
**Parathyroid hormone** (RR: 15-65 pg/mL)	167.1 (108.7)	65.3 (27.9)	<0.001^‡^
**25-hydroxyvitamin D_3_** (RR: 30-80 μg/L)	17.6 (14.3)	20.8 (6.53)	0.036^‡^
**Estimated glomerular filtration rate** (mL/min/1.73 m^2^)	97.7 ± 19.5	95.5 ± 19.8	0.1^†^

*SD – standard deviation; IQR – interquartile range; RR – reference range.

†t-test.

‡Wilcoxon’s test for paired samples.

The low eGFR group and normal eGFR group did not significantly differ in sex, nephrolithiasis rate, and DM rate. However, the low eGFR group had a significantly higher hypertension rate (*P* = 0.017) and was significantly older (*P* < 0.001). The low eGFR group also had significantly higher pre-parathyroidectomy 25(OH)D_3,_ pre-parathyroidectomy calcium, and post-parathyroidectomy creatinine, and significantly lower post-parathyroidectomy eGFR (Table 2). In the normal eGFR group, eGFR was significantly reduced after parathyroidectomy (*P* = 0.005) but in the low eGFR group it did not change significantly (*P* = 0.522, paired sample *t* test, data not shown). There were 4 patients with unchanged, 22 patients with decreased, and 45 patients with increased postoperative eGFR levels.

**Table 2:** Clinical data of primary hyperparathyroidism patients subdivided according to preoperative estimated glomerular filtration rate (eGFR)*

	Low eGFR (60≤eGFR<90) (n = 23)	Normal eGFR (eGFR≥90) (n = 48)	P
**Females, n (%)**	21 (91.3)	44 (91.7)	0.959^§^
**Hypertension, n (%)**	14 (60.9)	15 (31.3)	0.017^§^
**Diabetes mellitus, n (%)**	5 (21.7)	7 (14.6)	0.451^§^
**Nephrolithiasis, n (%)**	6 (26.1)	7 (14.6)	0.241^§^
**Age (years), mean±SD**	62.8 ± 8.1	48.0 ± 10.7	<0.001^†^
**Before parathyroidectomy**			
**Serum creatinine** (mg/dL), **mean±SD**	0.85 ± 0.12	0.62 ± 0.11	<0.001^†^
**Serum calcium** (mg/dL), **mean±SD**	11.7 ± 0.9	11.2 ± 0.6	0.012^†^
**Parathyroid hormone** (pg/mL), **median (IQR)**	154.4 (69.1)	179 (116.0)	0.589^‡^
**25-hydroxyvitamin D_3_** (μg/L), **median (IQR)**	20.7 (12.8)	14.8 (17.0)	0.033^‡^
**eGFR** (mL/min/1.73 m^2^), **mean±SD**	75.4 ± 9.8	108.4 ± 12.7	<0.001^†^
**After parathyroidectomy**			
**Serum creatinine** (mg/dL), **mean±SD**	0.86 ± 0.19	0.66 ± 0.13	<0.001^†^
**Serum calcium** (mg/dL), **mean±SD**	9.5 ± 0.4	9.5 ± 0.4	0.852^†^
**Parathyroid hormone** (pg/mL), **median (IQR)**	78.9 (54.3)	64.2 (27.4)	0.094^‡^
**25-hydroxyvitamin D_3_** (μg/L), **median (IQR)**	22.8 (17.0)	20.4 (6.4)	0.273^‡^
**eGFR** (mL/min/1.73 m^2^), **mean±SD**	77.2 ± 16.8	104.3 ± 14.6	<0.001^†^

*SD – standard deviation; IQR – interquartile range.

†t-test.

‡Mann-Whitney-U test.

§χ^2^ test.

The low calcium group and high calcium group did not significantly differ in sex and DM rate. The high calcium group had significantly higher mean age, nephrolithiasis rate, and hypertension rate. It also had significantly higher creatinine and calcium levels both before and after parathyroidectomy, and significantly lower pre-parathyroidectomy eGFR (Table 3). In the low calcium group, eGFR did not change after parathyroidectomy (*P* = 0.850), but in the high calcium group it was significantly reduced (*P* = 0.022) (paired sample *t* test, not shown in the table).

**Table 3:** Clinical data of primary hyperparathyroidism patients subdivided according to serum calcium

	Low serum calcium (≤11.2 mg/dL) (n = 35)	High serum calcium (>11.2 mg/dL) (n = 36)	P
**Female sex, n (%)**	33 (94.3)	32 (88.9)	0.414^§^
**Hypertension, n (%)**	8 (22.9)	21 (58.3)	**0.02**^§^
**Diabetes mellitus, n (%)**	3 (8.6)	9 (25)	0.065^§^
**Nephrolithiasis, n (%)**	2 (5.7)	11 (30.6)	**0.007**^§^
**Age (years), mean±SD**	48.7 ± 12.6	56.8 ± 10.3	**0.004^†^**
**Before parathyroidectomy**			
**Serum creatinine** (mg/dL), **mean±SD**	0.64 ± 0.16	0.74 ± 0.14	**0.006^†^**
**Serum calcium** (mg/dL), **mean±SD**	10.8 ± 0.38	11.9 ± 0.59	**<0.001^†^**
**Parathyroid hormone** (pg/mL), **median (IQR)**	141.3 (124.1)	182.0 (83.1)	0.182^‡^
**25-hydroxyvitamin D_3_** (μg/L), **median (IQR)**	16.5 (10.6)	19.3 (18.5)	0.412^‡^
**eGFR** (mL/min/1.73 m^2^), **mean±SD**	105.1 ± 19.1	90.5 ± 17.3	**0.001^†^**
**After parathyroidectomy**			
**Serum creatinine** (mg/dL), **mean±SD**	0.65 ± 0.14	0.79 ± 0.19	**0.001^†^**
**Serum calcium** (mg/dL), **mean±SD**	9.38 ± 0.42	9.64 ± 0.37	**0.007^†^**
**Parathyroid hormone** (pg/mL), **median (IQR)**	65.2 (25.9)	65.3 (40.5)	0.321^‡^
**25-hydroxyvitamin D_3_** (μg/L), **median (IQR)**	22.6 (5.9)	20.5 (9.9)	0.291^‡^
**eGFR** (mL/min/1.73 m^2^), **median (IQR)**	104.0 (27)	86.5 ± 19.1	**<0.001^†^**

*SD – standard deviation; IQR – interquartile range.

†t-test.

‡Mann-Whitney-U test. **·**

§χ^2^ test.

Pre-parathyroidectomy eGFR negatively correlated with age, pre-parathyroidectomy calcium, creatinine, and 25(OH)D_3_ whereas post-parathyroidectomy eGFR negatively correlated with age, and pre-parathyroidectomy calcium and creatinine (Table 4).

**Table 4:** Correlation between laboratory parameters and pre- and post-parathyroidectomy estimated glomerular filtration rate (eGFR)

	Pre-parathyroidectomy					
	eGFR	age	creatinine	calcium	parathyroid hormone	25-hydroxyvitamin D_3_
Pre-parathyroidectomy eGFR						
correlation coefficient	-	-0.760*	-0.888*	-0.290^†^	0.168	-0.296^†^
significance (two-tailed)	-	<0.001^‡^	<0.001^‡^	0.014^‡^	0.161^§^	0.027^§^
Post- parathyroidectomy eGFR						
correlation coefficient	0.835*	-0.717*	-0.739*	-0.385*	0.079	-0.255
significance (two-tailed)	<0.001^‡^	<0.001^‡^	<0.001^‡^	0.001^‡^	0.514^§^	0.058^§^

*Correlation is significant at the 0.01 level (two-tailed).

†Correlation is significant at the 0.05 level (two-tailed).

‡Pearson correlation.

§Spearman correlation.

Regression analysis assessed the impact of hypertension, DM, age, baseline serum calcium, creatinine, eGFR, and PTH levels on eGFR changes. PTH, hypertension, or DM did not significantly affect eGFR change. The most significant model (including the variables of age, baseline serum calcium, creatinine and eGFR levels) was Model F = 4.276; model R^2^ = 0.206; *P* = 0.004 (Table 5). All these variables, except baseline serum creatinine, were independent predictors of eGFR change.

**Table 5:** Multiple linear regression model of predictors of estimated glomerular filtration rate (eGFR) change between pre- and post-parathyroidectomy values*

Independent variables	Beta	P
**Age, years**	0.531	**0.021**
**Serum creatinine, mg/dL**	0.523	**0.101**
**Serum calcium, mg/dL**	0.251	**0.034**
**eGFR (mL/min/1.73 m^2^)**	1.202	**0.006**

*Dependent variable: eGFR change (continuous variable), R^2^ of the model = 0.206, *P* = 0.004.

## Discussion

This study points to an association between mild renal dysfunction in PHPT and older age, hypertension, and high serum calcium levels. In addition, older age, high basal eGFR, and high basal calcium levels were associated with a decrease in eGFR after surgery. Our findings are in accordance with those reported in previous research, which also found an association between low eGFR and hypertension, older age, and high calcium levels, although these studies included patients with lower eGFR (1,4).

The most recent guideline on the management of asymptomatic PHPT confirmed 60 mL/min/1.73 m^2^ as the renal impairment threshold for parathyroidectomy, which is equivalent to stage 3 chronic kidney disease. Below this level, decreased renal function may negatively affect PTH levels (16,17). However, some studies reported secondary increases in PTH levels and PHPT exacerbation at an eGFR threshold ranging from 30 to 70 mL/min/1.73 m^2^ (17-19). These differences might be explained by the methodological differences and different number of participants between the studies. The present study included patients with eGFRs ≥60 mL/min/1.73 m^2^ to eliminate the impact of renal dysfunction on PTH levels.

Several previous studies investigated the effects of parathyroidectomy on renal impairment. In patients with decreased renal function (eGFR<60 mL/min/1.73 m^2^), parathyroidectomy did not improve eGFR (6-12,19-21) but it did halt eGFR deterioration (4,5,19). Our study showed for the first time that patients with eGFR between 60 and 90 mL/min/1.73 m^2^ had a preserved renal function, whereas those with eGFR≥90 mL/min/1.73 m^2^ had a decreased eGFR in post-parathyroidectomy period.

Nair et al (4) reported an increased prevalence of renal dysfunction when calcium levels exceeded 12 mg/dL. The risk of developing renal failure was increased 13.8-fold even in mild PHPT (mean calcium: 10.5 mg/dL) (22). To the best of our knowledge, this is the first study to evaluate the impact of pre-surgery calcium levels on pre- and post-surgery eGFR by dividing the patients according to their calcium levels. The results revealed that the high calcium group had significantly low eGFR and more common renal injury after parathyroidectomy than the low calcium group. This finding may be explained by the presence of PTH, a potent vasodilator of renal arteries and arterioles. Therefore, increased PTH levels in PHPT may lead to increased glomerular flow and increased GFR (14,23-25). In previous studies, increased pre-glomerular resistance after abrupt parathyroidectomy-related decreases in PTH levels was associated with acute renal injury (14,24-26). Although we found no association between PTH levels and eGFR change 3-6 months after surgery, calcium levels were related to eGFR changes.

The post-parathyroidectomy eGFR decrease was more frequent in the normal eGFR group and high calcium group. This is not a contradiction because, despite parathyroidectomy, calcium remained higher in the high calcium group than in the low calcium group. Also, there were more patients in the high calcium group with a decrease in their eGFR postoperatively most probably due to the effect of abruptly falling PT. There was no difference in calcium levels between the low eGFR group and the normal eGFR group in the postoperative period.

The known causes of renal dysfunction include hypertension, DM, and renal complications. Some studies reported a significantly high prevalence of DM and hypertension among PHPT patients (4,27,28). In our study, there was no significant difference between the groups in the DM rate. In addition, the patients were evaluated as soon as 3-6 months after surgery to minimize the effects of chronic disease processes such as DM and hypertension on eGFR. A longer follow-up interval after surgery might have provided misleading information on eGFR changes. Some previous studies observed transient changes in renal function in patients with PHPT in the first 48 hours after parathyroidectomy, which continued for up to 6 months (22,29). In our patients, there were no major surgical complications, such as severe hypotension. Therefore, the time interval in the present study was long enough to allow renal recovery after surgery.

Most previous studies that evaluated the association between PHPT and eGFR did not classify the patients according to their pathological diagnosis (parathyroid adenoma or hyperplasia). We included only patients with parathyroid adenoma. Although there are several studies on PHPT and eGFR, no other study evaluated the eGFR changes after parathyroidectomy according to pre-operative calcium levels. We believe that this study elucidates the effects of parathyroidectomy and pre-operative hypercalcemia on eGFR more clearly in a specific group of patients with parathyroid adenoma and eGFR level ≥60 mL/min/1.73 m^2^.

Our study has several limitations, most notably its retrospective design and short evaluation time. In all the eGFR calculations, we used a single creatinine level, which may not reflect the actual eGFR. Our sample size was limited because of our strict inclusion criteria. Further studies on the effects of parathyroidectomy in a larger group of patients with eGFRs ≥60 mL /min/1.73 m^2^ are needed to confirm our results and further explain the etiology of renal dysfunction in PHPT and eGFR changes post-surgery.

In conclusion, we believe that these data provide new insight into the effect of hypercalcemia and parathyroidectomy on pre- and post-parathyroidectomy eGFR levels. Mild renal dysfunction in PHPT was associated with older age, hypertension, and high serum calcium levels. Age and pre-parathyroidectomy eGFR and calcium levels were predictors of eGFR changes after surgery. In addition, older age, high baseline eGFR, and high baseline calcium levels were associated with eGFR decrease after surgery.

## References

[1] Walker MD, Nickolas T, Kepley A, Lee JA, Zhang C, McMahon DJ (2014). Predictors of renal function in primary hyperparathyroidism.. J Clin Endocrinol Metab.

[2] Khan AA, Hanley DA, Rizzoli R, Bollerslev J, Young JE, Rejnmark L (2017). Primary hyperparathyroidism: review and recommendations on evaluation, diagnosis, and management. A Canadian and international consensus.. Osteoporos Int.

[3] Corbetta S, Baccarelli A, Aroldi A, Vicentini L, Fogazzi GB, Eller-Vainicher C (2005). Risk factors associated to kidney stones in primary hyperparathyroidism.. J Endocrinol Invest.

[4] Nair CG, Babu M, Jacob P, Menon R, Mathew J (2016). Unnikrishnan. Renal dysfunction in primary hyperparathyroidism; effect of parathyroidectomy: a retrospective cohort study.. Int J Surg.

[5] Tassone F, Guarnieri A, Castellano E, Baffoni C, Attanasio R, Borretta G (2015). Parathyroidectomy halts the deterioration of renal function in primary hyperparathyroidism.. J Clin Endocrinol Metab.

[6] Ghose RR, Morgan WD (1981). Improvement in renal function in primary hyperparathyroidism following parathyroidectomy.. Postgrad Med J.

[7] Valdemarsson S, Lindergĺrd B, Tibblin S, Bergenfelz A (1998). Increased biochemical markers of bone formation and resorption in primary hyperparathyroidism with special reference to patients with mild disease.. J Intern Med.

[8] Jones DB, Jones JH, Lloyd HJ, Lucas PA, Wilkins WE, Walker DA (1983). Changes in blood pressure and renal function after parathyroidectomy in primary hyperparathyroidism.. Postgrad Med J.

[9] Salahudeen AK, Thomas TH, Sellars L, Tapster S, Keavey P, Farndon JR (1989). Hypertension and renal dysfunction in primary hyperparathyroidism: effect of parathyroidectomy.. Clin Sci (Lond).

[10] Falkheden T, Ohlsson L, Sjögren B (1980). Renal function in primary hyperparathyroidism. A follow-up study two to eleven years after surgery comprising 139 patients.. Scand J Urol Nephrol.

[11] Hedbäck G, Abrahamsson K, Odén A (2001). The improvement of renal concentration capacity after surgery for primary hyperparathyroidism.. Eur J Clin Invest.

[12] Hendrickson CD, Castro Pereira DJ, Comi RJ (2014). Renal impairment as a surgical indication in primary hyperparathyroidism: do the data support this recommendation?. J Clin Endocrinol Metab.

[13] Tassone F, Gianotti L, Emmolo I, Ghio M, Borretta G (2009). Glomerular filtration rate and parathyroid hormone secretion in primary hyperparathyroidism.. J Clin Endocrinol Metab.

[14] Santos JHZ, Enout MJR, Shimozono AT, Fujjiz LI, Rocha LA, Santos RO (2018). Evolution of renal function in patients with primary hyperparathyroidism submitted to parathyroidectomy.. Arch Head Neck Surg..

[15] (2013). KDIGO 2012 clinical practice guideline for the evaluation and management of chronic kidney disease.. Kidney Int Suppl.

[16] Bilezikian JP, Brandi ML, Eastell R, Silverberg SJ, Udelsman R, Marcocci C (2014). Guidelines for the management of asymptomatic primary hyperparathyroidism: summary statement from the Fourth International Workshop.. J Clin Endocrinol Metab.

[17] Gianotti L, Tassone F, Cesario F, Pia A, Razzore P, Magro G (2006). A slight decrease in renal function further impairs bone mineral density in primary hyperparathyroidism.. J Clin Endocrinol Metab.

[18] Yamashita H, Noguchi S, Uchino S, Watanabe S, Murakami T, Ogawa T (2003). Influence of renal function on clinico-pathological features of primary hyperparathyroidism.. Eur J Endocrinol.

[19] Kristoffersson A, Backman C, Granqvist K, Järhult J (1990). Pre- and postoperative evaluation of renal function with five different tests in patients with primary hyperparathyroidism.. J Intern Med.

[20] Posen S, Clifton-Bligh P, Reeve TS, Wagstaffe C, Wilkinson M (1985). Is parathyroidectomy of benefit in primary hyperparathyroidism?. Q J Med.

[21] Rowlands C, Zyada A, Zouwail S, Joshi H, Stechman MJ, Scott-Coombes DM (2013). Recurrent urolithiasis following parathyroidectomy for primary hyperparathyroidism.. Ann R Coll Surg Engl.

[22] Yu N, Donnan PT, Leese GP (2010). A record linkage study of outcomes in patients with mild primary hyperparathyroidism: the Parathyroid Epidemiology and Audit Research Study (PEARS).. Clin Endocrinol (Oxf).

[23] Parikh S, Nagaraja H, Agarwal A, Samavedi S, Von Visger J, Nori U (2013). Impact of post-kidney transplant arathyroidectomy on allograft function.. Clin Transplant.

[24] Endlich K, Massfelder T, Helwig JJ, Steinhausen M (1995). Vascular effects of parathyroid hormone and parathyroid hormone-related protein in the split hydronephrotic rat kidney.. J Physiol.

[25] Clemens TL, Cormier S, Eichinger A, Endlich K, Fiaschi-Taesch N, Fischer E (2001). Parathyroid hormone-related protein and its receptors: nuclear functions and roles in the renal and cardiovascular systems, the placental trophoblasts and the pancreatic islets.. Br J Pharmacol.

[26] Peacock M (2002). Primary hyperparathyroidism and the kidney: biochemical and clinical spectrum.. J Bone Miner Res.

[27] Cardenas MG, Vigil KJ, Talpos GB, Lee MW, Peterson E, Rao DS (2008). Prevalence type 2 diabetes mellitus in patients with primary hyperparathyroidism.. Endocr Pract.

[28] Rosenthal FD, Roy S (1972). Hypertension and hyperparathyroidism.. BMJ.

[29] Montenegro FLM, Martin RM, Lourenço DM, Arap SS, Nascimento CP, Brandăo LG (2010). Alteraçăo transitória da taxa de filtraçăo glomerular após paratireoidectomia independe da funçăo renal pré-operatória.. Rev Bras Cir Cabeça Pescoço..

